# Supplementary material for: Revised Minoan eruption volume as benchmark for large volcanic eruptions
Source: Nat Commun. 2023 Apr 29;14:2497. doi: 10.1038/s41467-023-38176-3 (PMC10148807; doi:10.1038/s41467-023-38176-3)
Supplement: Supplementary file 1 — Supplementary Information [file 41467_2023_38176_MOESM1_ESM.pdf]

Supplementary Information

Supplementary Figure 1

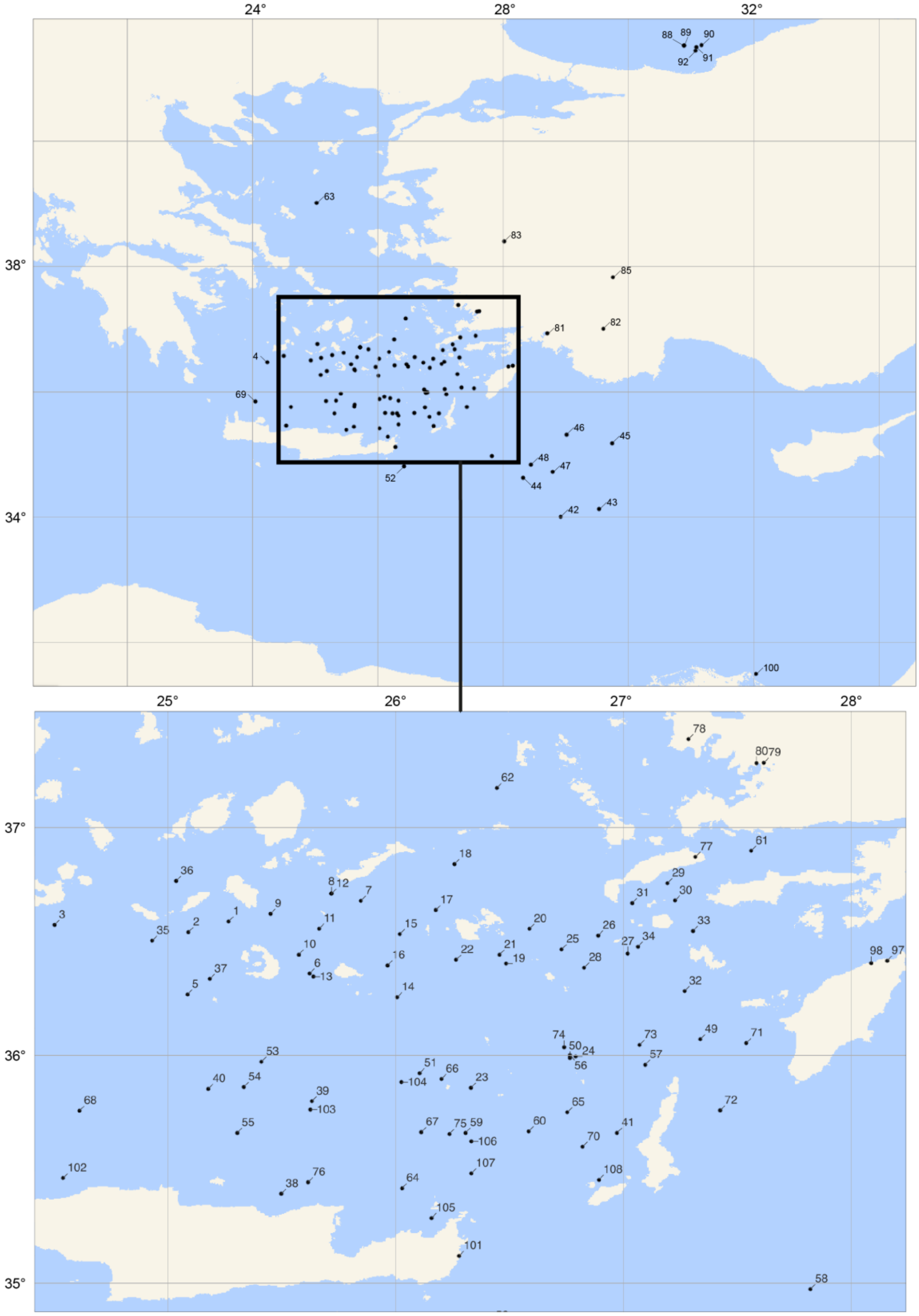

Figure S1 | Map showing locations of Minoan tephra thickness measurements (numbers refer to Map-ID in Supplementary Dataset 2).

Supplementary Figure 2

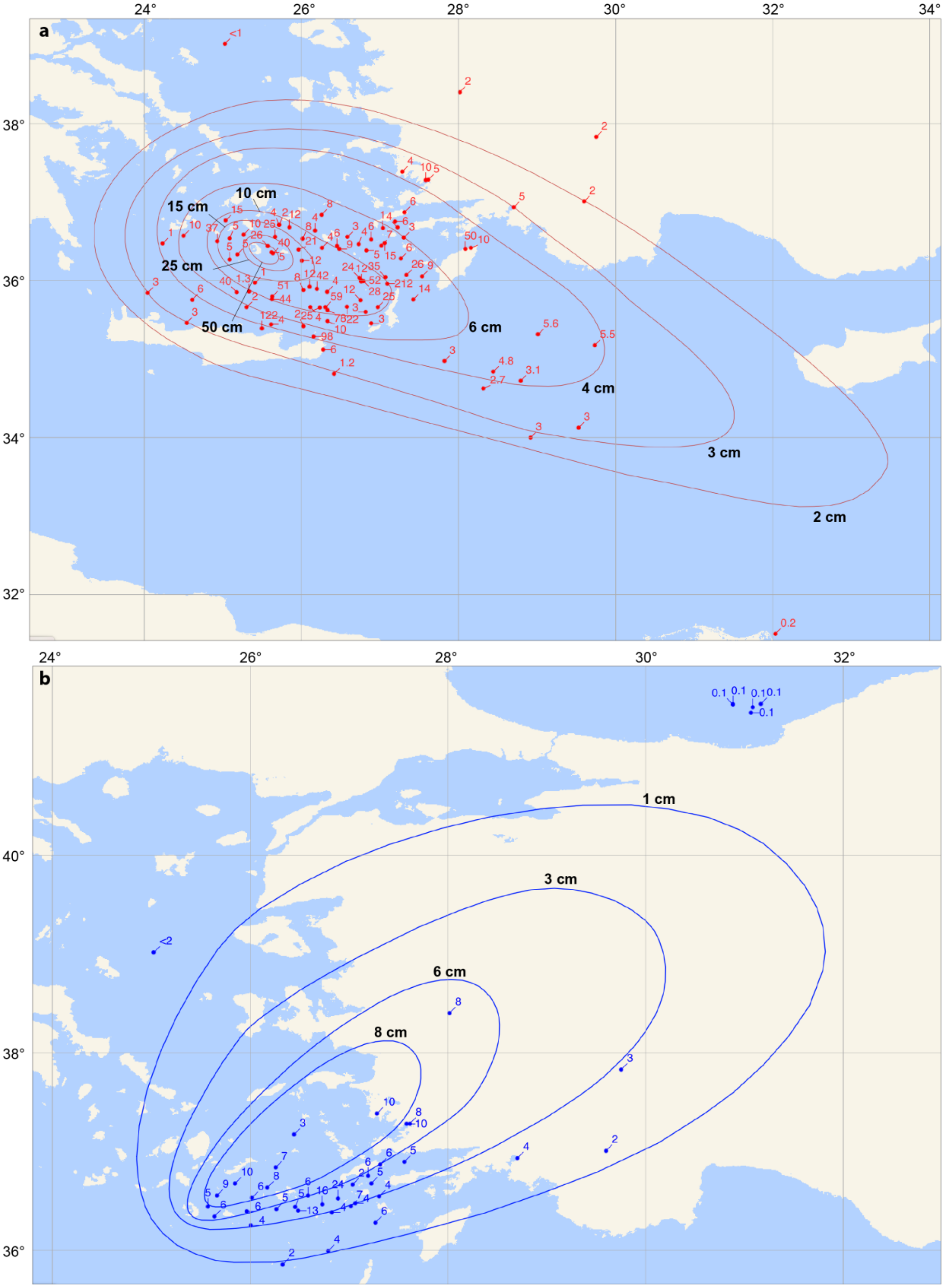

Figure S2 | **a** Plinian phase isopachs based on tephra thickness measurements. **b** Co-ignimbrite phase isopachs based on tephra thickness measurements.

Supplementary Note 1 – Sediment core analysis

The tripartite nature of core POS513-20 is also present in the 66-cm-thick Minoan deposits in core POS513-41, obtained ~140 km from the eruptive centre (**Fig. S3a)**. Due to the greater distance from source, grain size is generally smaller, and individual beds are thinner than in POS513-20. The lower subunit shows normally graded beds of medium tephra overlain by a more massive sequence with faint lamination. The boundary of the lower subunit to the underlying hemipelagic sediments is unconformable (**Fig. S3d**), which is the result of the comparably dense tephra material sinking into the substrate causing the development of a flame structure. As in core POS513-20, prominent colour and grayscale changes mark the boundary to the middle subunit, which indicate a higher lithic and crystal content and an increase in grain size (**Fig. S3c**). Cross-lamination characterizes the fabric of the middle section and the boundary to the upper unit is defined by a transition to structureless material (of similar grain size) in the upper unit (**Fig. S3c**), which consists of fine ash. Again, the diffuse transition to hemipelagic sediments is indicated by porosity change (**Fig. S3a**).

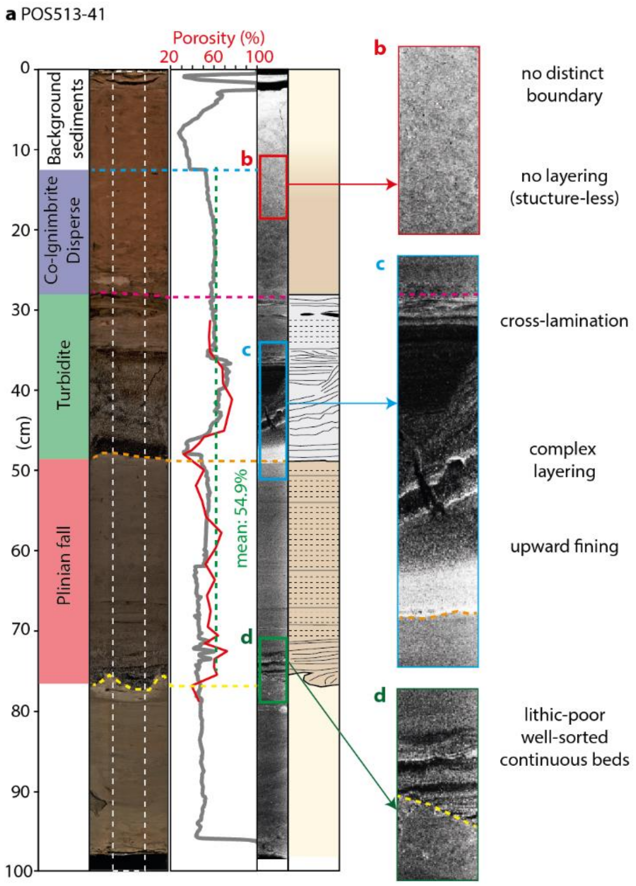

Figure S3 | Sedimentological analysis of marine gravity core POS513-41. (**a**) Photographic scans, porosity measurements, X-ray CT-scan and stratigraphic interpretation. (**b**) - (**d**) Enlargements of key intervals of the X-ray CT-scan showing differences between the deposit subunits.

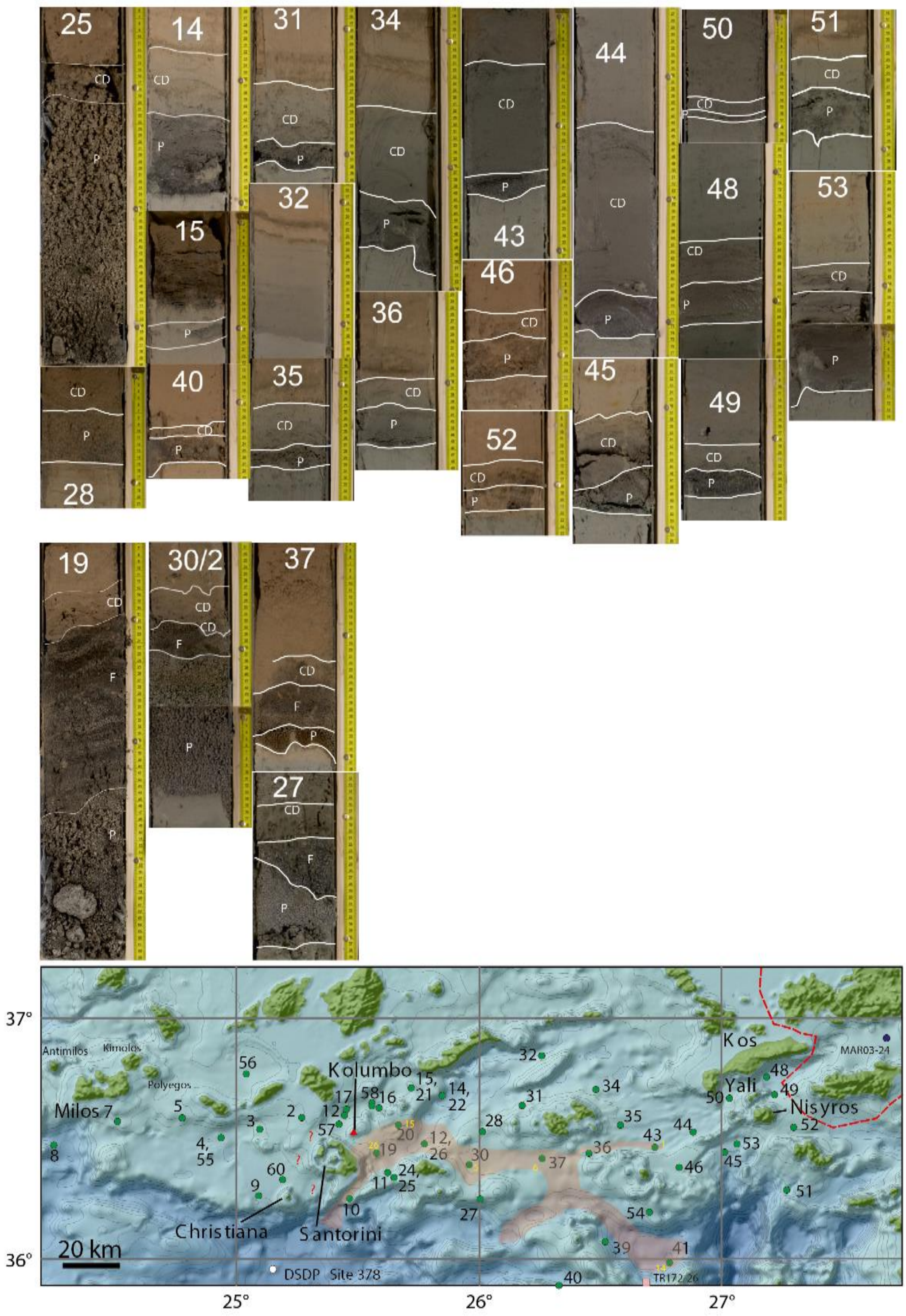

Figure S4 | Sediment cores from the Southern Aegean Sea. Upper panel shows interpreted core photographs indicating Plinian tephra fall deposits (P) and co-ignimbrite tephra fall deposits/dispersed tephra portion (CD) from marine Minoan tephra in selected cores east of Santorini from research cruise POS513. Middle panel shows the interpreted core pictures indicating tripartition of the marine Minoan tephra sequence into basal Plinian fall deposits (P), middle flow-related deposits (F), and co-ignimbrite tephra fall deposits/dispersed tephra portion (CD) from selected cores east of Santorini from research cruise POS513. Lower panel indicates the position of all POS 513 cores that show tripartition of the marine Minoan tephra sequence and respective thickness in cm of the turbidite unit (yellow numbers)

as well as the estimated areal distribution of the flow deposits following bathymetric depressions. Red question marks indicate areas, where no cores could be recovered.

Supplementary Note 2 - Correlation between CT-grayscale value analysis and measured porosity

As seen in **Figs. 1 and S3**, both CT-derived and physically measured density estimates capture the same trends in the scanned sections of POS513-20 and POS513-41. Based on the homogenous geochemistry and solid density reported for both Minoan tephra deposits by Kutterolf et al. (**refs. 1, 2**), we attribute these variations to down-core porosity changes. This interpretation is further supported by the strong anti-correlation (R = -0.64, p = 0.0003, n= 34) between density and particle size measured on samples from both investigated cores (see methods). We show the 95% confidence interval in Fig. S5.

Following from the above, we argue that our down-core CT grayscale data can be used to revise MT tephra fall volume estimates by refining density and porosity calculations. To do so, we i) harness the afore-mentioned relation between CT-derived and physically measured density estimates to model density variations at 500 µm down-core intervals as shown in Supl. Fig. S5, ii) transform these to porosity estimates using the Minoan tephra solid density values reported by Kutterolf et al. (**refs. 1, 2**) and the formula by Caffrey (**ref. 3**): porosity = 1 – (bulk density/solid density), and 3) validate this approach using independent CT-derived pore volume estimates (see methods). The latter effort is, however, hampered by scanning resolution limits that prevent us from resolving pores smaller than 100 µm (see methods). While this drawback greatly affects CT porosity estimates for the distal fine-grained ash in POS 513-41, the results for the sand-sized MT deposit in proximal POS 513-20 are far more encouraging and support the validity of our approach (**Fig. S5**).

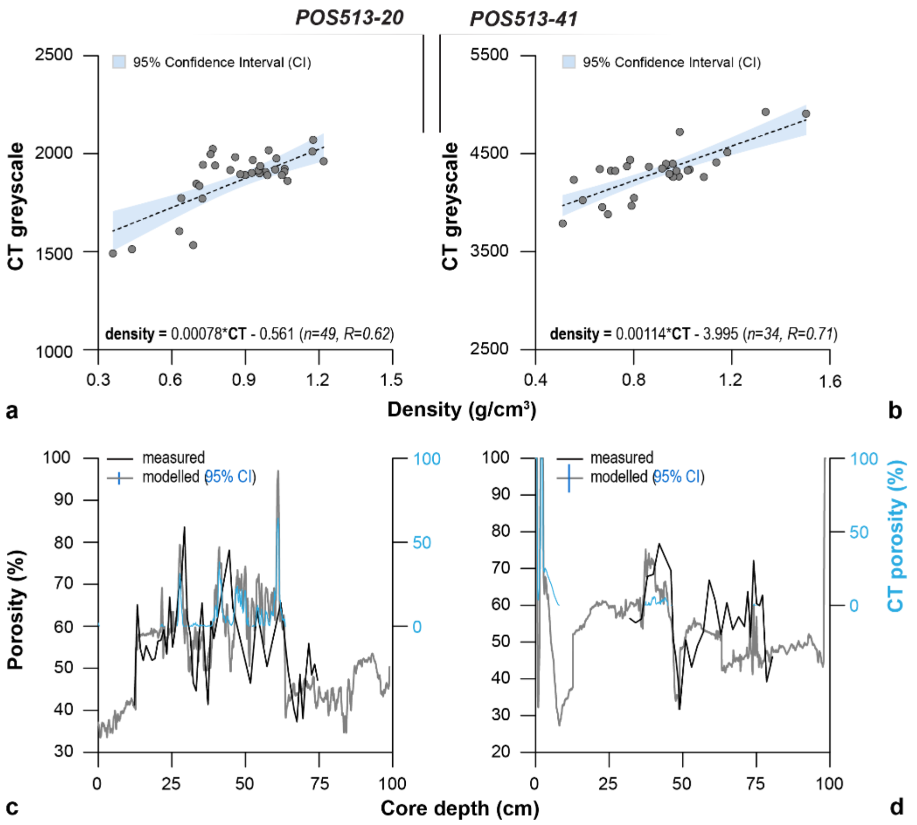

Figure S5 | Correlation between CT-grayscale value analysis and measured porosity. (**a**) and (**b**) Plots of CT greyscale values against density for sediment cores POS513-20 and POS513-41. (**c**) and (**d**) Measured and modelled porosities for sediment cores POS513-20 and POS513-41.

Supplementary References

1. Kutterolf, S., et al. The medial offshore record of explosive volcanism along the central to eastern Aegean Volcanic Arc: 1. Tephra ages and volumes, eruption magnitudes and marine sedimentation rate variations. Geochemistry, Geophysics, Geosystems, 22(12), e2021GC010010 (2021).
2. Kutterolf, S., et al. The medial offshore record of explosive volcanism along the central to eastern Aegean Volcanic Arc: 2. Tephra ages and volumes, eruption magnitudes and marine sedimentation rate variations. Geochemistry, Geophysics, Geosystems, 22(12), e2021GC010011 (2021).
3. Caffrey, J. M. Spatial and seasonal patterns in sediment nitrogen remineralization and ammonium concentrations in San Francisco Bay, California. Estuaries, 18(1), 219-233 (1995).
