# Supplementary material for: Revised Minoan eruption volume as benchmark for large volcanic eruptions
Source: Nat Commun. 2023 Apr 29;14:2497. doi: 10.1038/s41467-023-38176-3 (PMC10148807; doi:10.1038/s41467-023-38176-3)
Supplement: Supplementary file 3 — Description of Additional Supplementary Files [file 41467_2023_38176_MOESM3_ESM.pdf]

Description of Additional Supplementary Files

File name: Supplementary Data 1

Description: CT-grayscale value analysis results

File name: Supplementary Data 2

Description: Marine sediment cores used for isopach analysis
